# Fracture of Cranium; Hernia Cerebri; Recovery

**Published:** 1887-09

**Authors:** William Henry

**Affiliations:** Harmon, Illinois


### FRACTURE OF CRANIUM; HERNIA CEREBRI; RECOVERY.

BY WILLIAM HENRY, M. D., OF HARMON, ILL.

In June, 1886, my little daughter, aged 8 years, while trying to drive a horse running at large, was kicked on the side of the head, the skull being fractured over the posterior superior portion of the right parietal bone, crushing the bone and driving it in, producing compression of the brain.

I was absent from home at the time, and my friend, Dr. Burns, was called in; his opinion was that she would not live twelve hours. I was immediately telegraphed for, and on arriving at home in a few hours, I found the child in a comatose condition, her pulse regular and about 110. I sent for Dr. Burns, and we soon went to work to remove the compressed bone, but after the removal of seven pieces of bone the comatose condition was not entirely relieved. She remained in a semi-comatose condition for five days.

We found the membranes ruptured in the anterior portion of the fracture, and portions of cerebrum oozing therefrom; in all, about half an ounce was lost. The edges of the wound were united with stitches, the antiseptic used being a dilute solution of carbolic acid. In a short time the wound began to suppurate, and became very much swollen. In the meantime I had returned to my place of business, leaving the patient in charge of Dr. Burns, who sent for me again when this last complication arose. I thought it would be best to open the wound in the lowest portion and let out the pus. On his doing so a considerable amount of pus escaped.

I again returned to my place of business, and in a short time Dr. Burns thought that the swollen condition was increasing, and cut the stitches. This caused the wound to gape open, and there soon appeared a hernia cerebri, about as large as a hen's egg. Seeing this condition, Dr. Burns called in Dr. Helm, of Rockford, and Drs. Donaldson and Herb; but they hesitated to advise active interference during my absence. On my return, in a few days, I found the hernia cerebri still increasing, and after a consultation with Drs. Burns and Donaldson, I determined to ligate the tumor at its base. I accordingly tied it with silk ligature, drawing the ligature until the pulsation was almost diminished; and in a few days I again tied it, drawing the ligature until all pulsation ceased. It then began to heal under the ligature as the strangulated portion began to die and dry up; and in about fifteen days the tumor fell off, leaving the wound closed and nicely healed.—*Journal of the American Medical Association.*

